# Influenza A virus enhances ciliary activity and mucociliary clearance via TLR3 in airway epithelium

**DOI:** 10.1186/s12931-020-01555-1

**Published:** 2020-10-27

**Authors:** Yosuke Kamiya, Tomoyuki Fujisawa, Mineo Katsumata, Hideki Yasui, Yuzo Suzuki, Masato Karayama, Hironao Hozumi, Kazuki Furuhashi, Noriyuki Enomoto, Yutaro Nakamura, Naoki Inui, Mitsutoshi Setou, Masahiko Ito, Tetsuro Suzuki, Koji Ikegami, Takafumi Suda

**Affiliations:** 1 Second Division, Department of Internal Medicine, Hamamatsu University School of Medicine, 1-20-1 Handayama, Higashi-ku, Hamamatsu, Shizuoka 431-3192 Japan; 2 Department of Clinical Pharmacology and Therapeutics, Hamamatsu University School of Medicine, 1-20-1 Handayama, Higashi-ku, Hamamatsu, 431-3192 Japan; 3 Department of Cellular and Molecular Anatomy and International Mass Imaging Center, Hamamatsu University School of Medicine, 1-20-1 Handayama, Higashi-ku, Hamamatsu, 431-3192 Japan; 4 Department of Virology and Parasitology, Hamamatsu University School of Medicine, 1-20-1 Handayama, Higashi-ku, Hamamatsu, 431-3192 Japan; 5 Department of Anatomy and Developmental Biology, Graduate School of Biomedical and Health Sciences, Hiroshima University, 1-2-3 Kasumi, Minamiku, Hiroshima 734-8553 Japan

**Keywords:** Influenza A virus, TLR3, Ciliary activity, Mucociliary clearance, Airway epithelium

## Abstract

**Background:**

Viral respiratory tract infections, such as influenza A virus (IAV), are common and life-threatening illnesses worldwide. The mechanisms by which viruses are removed from the respiratory tract are indispensable for airway host defense. Mucociliary clearance is an airway defense mechanism that removes pathogens from the respiratory tract. The coordination and modulation of the ciliary beating of airway epithelial cells play key roles in maintaining effective mucociliary clearance. However, the impact of respiratory virus infection on ciliary activity and mucociliary clearance remains unclear.

**Methods:**

Tracheal samples were taken from wild-type (WT) and Toll-like receptor 3 (TLR3)-knockout (KO) mice. Transient organ culture of murine trachea was performed in the presence or absence of IAV, polyI:C, a synthetic TLR3 ligand, and/or reagents. Subsequently, cilia-driven flow and ciliary motility were analyzed. To evaluate cilia-driven flow, red fluorescent beads were loaded into culture media and movements of the beads onto the tracheal surface were observed using a fluorescence microscope. To evaluate ciliary motility, cilia tips were labeled with Indian ink diluted with culture medium. The motility of ink-labeled cilia tips was recorded by high-speed cameras.

**Results:**

Short-term IAV infection significantly increased cilia-driven flow and ciliary beat frequency (CBF) compared with the control level in WT culture. Whereas IAV infection did not elicit any increases of cilia-driven flow and CBF in TLR3-KO culture, indicating that TLR3 was essential to elicit an increase of cilia-driven flow and CBF in response to IAV infection. TLR3 activation by polyI:C readily induced adenosine triphosphate (ATP) release from the trachea and increases of cilia-driven flow and CBF in WT culture, but not in TLR3-KO culture. Moreover, blockade of purinergic P2 receptors (P2Rs) signaling using P2R antagonist, suramin, suppressed polyI:C-mediated increases of cilia-driven flow and CBF, indicating that TLR3-mediated ciliary activation depended on released extracellular ATP and the autocrine ATP-P2R loop.

**Conclusions:**

IAV infection readily increases ciliary activity and cilia-driven flow via TLR3 activation in the airway epithelium, thereby hastening mucociliary clearance and “sweeping” viruses from the airway as an initial host defense response. Mechanically, extracellular ATP release in response to TLR3 activation promotes ciliary activity through autocrine ATP-P2R loop.

## Background

Viral respiratory tract infections by RNA viruses (e.g., influenza A virus [IAV], rhinovirus, coronavirus, and respiratory syncytial virus) are among the most common illnesses worldwide, and their severity widely varies from the common cold to severe respiratory tract infections [1–3]. For instance, IAV causes seasonal respiratory infection, leading to half a million deaths annually. Only a few clinically effective vaccines or specific antiviral drugs are available for the prevention and treatment of viral respiratory infections. Thus, the rational mechanisms by which viruses are removed from the respiratory tract are indispensable for achieving effective viral clearance and airway host defense. Viral respiratory infections are also major causes of the acute exacerbation of chronic airway diseases, such as asthma and chronic obstructive pulmonary disease [4, 5].

The airway epithelium acts as a frontline defense against foreign substances, including viruses, bacteria, and environmental air pollution, serving both as a physical barrier and a regulator of innate and adaptive immune responses [6–9]. Mucociliary clearance is an important defense mechanism in the respiratory tract that requires coordinated ciliary activity and proper mucus production to propel airway surface liquids that traps pathogens and pollutants, permitting their clearance from the lungs [10, 11]. Ciliated cells, with a large number of cilia on the luminal side, represent the major cell type of the airway epithelium, and they play a central role in mucociliary clearance through the coordination and modulation of ciliary beating [10–12]. Ciliary beat frequency (CBF) is a major determinant of mucociliary clearance. To properly generate cilia-driven flow, the vigorous asymmetric beating of the cilia, which can be represented as effective and recovery strokes, are also required [13–15]. The importance of ciliary activity and mucociliary clearance in airway host defense is well documented in several diseases, such as primary ciliary dyskinesia and cystic fibrosis, in which impaired ciliary activity and mucociliary clearance predispose patients to recurrent airway infection [16, 17].

Previous studies revealed that extracellular components have the potential to enhance ciliary activity in the airway epithelium. Ciliated cells express bitter taste receptors. Bitter compounds (e.g., denatonium, thujone) stimulate these receptors and increase CBF in human airway epithelial cells, making ciliated cells chemosensory organelles [12]. The proinflammatory cytokines, tumor necrosis factor (TNF)-α and interleukin (IL)-1β, upregulate ciliary motility in a nitric oxide-dependent manner [18]. Arginine vasopressin, an antidiuretic hormone, rapidly increased CBF in rabbit tracheal epithelium by increasing intracellular Ca^2+^ levels [19]. In addition, CBF can be stimulated by several drugs used to treat pulmonary diseases, such as β-adrenergic agonists [20] and macrolide antibiotics [21].

The impact of respiratory virus infection on airway ciliary activity and mucociliary clearance remains to be elucidated. During respiratory viral infection, the interaction between viruses and airway epithelial cells through pattern-recognition receptors (PRRs) on the cell surface, such as Toll-like receptors (TLRs) [8, 22], results in the production of antiviral substances, including types I and III interferon (IFN), β-defensin, cytokines, and chemokines [23–28], which inhibit viral replication and mediate adaptive immunity responses. Recently, our group reported that IAV and polyI:C, a ligand of TLR3, increased the expression of IFN-λ and proinflammatory cytokines and chemokines (e.g., G-CSF, IL-8, IL-17C, CXCL1, CXCL5) in normal human bronchial epithelial cells [26–29], indicating that IAV-mediated TLR3 signaling plays pivotal roles in initial antiviral responses in the airway. However, no report has elucidated the relationships between TLR3 signaling and ciliary activity during viral infection, which is critical given that ciliary activity is a major determinant of the ability of mucociliary clearance to eliminate noxious viruses from the respiratory tract.

To more efficiently activate airway host defense mechanisms during respiratory viral infection, ciliary activity and mucociliary clearance should be activated in the airway. Given that airway epithelial cells produce antiviral substances and cytokines via virus-mediated TLR3 activation to promote protective immune responses, it is possible that viral infection exerts significant effects on ciliary activity and mucociliary clearance via TLR3 activation in the airway epithelium. In the present study, we investigated the effects of short-term IAV infection on ciliary activity and transport using an organ culture of murine tracheal tissue, an in vitro IAV infection model, and imaging techniques to analyze ciliary activity and cilia-driven flow. We also examined the roles of TLR3 activation in ciliary activity and cilia-driven flow during IAV infection to elucidate the regulatory mechanisms of IAV-mediated ciliary activation using tracheal epithelium from TLR3-knockout (KO) mice.

## Methods

### Mice

BALB/c mice were purchased from Japan SLC, Inc. (Shizuoka, Japan). TLR3-KO BALB/c mice were purchased from Oriental Bioservice, Inc. (Kyoto, Japan). All experiments involving mice followed protocols approved by the Animal Care and Use Committees of Hamamatsu University School of Medicine (license number H29-064) and were performed in accordance with the relevant guidelines and regulations.

### Isolation of murine trachea and organ culture of tracheal tissue

Tracheal samples were taken from 9 to 11-week-old female wild-type (WT) and TLR3-KO BALB/c mice and placed in cold collection medium solution (DMEM with sodium pyruvate solution) kept in an ice bath. Excess fat and connective tissue debris were immediately removed from the trachea using forceps, and subsequently, the membranous portion of the trachea was excised to expose the ciliated epithelium in the trachea. After the tracheal sample was treated, organ culture of murine trachea was performed in 2 mL of culture medium in the presence or absence of IAV, TLR ligand, and/or reagents in 35-mm culture dishes at 37 °C for 5–120 min. After organ culture of tracheal tissue, tracheal epithelium was observed and analyzed at room temperature (23–28 °C) using a dedicated microscope.

### Analysis of cilia-driven flow

Ciliary transport on the surface of intact trachea was analyzed after transient organ culture. To visualize cilia-driven flow, tracheal tissue was placed in a 35-mm culture dish with the luminal face down in 2 mL of culture medium containing 0.2-μm-diameter polystyrene beads (0.2-μm red fluorescent beads: Thermo Fisher Scientific, Waltham, MA, USA). Methylcellulose (M0512, Sigma-Aldrich, St. Louis, MO, USA) was added to the culture medium at a concentration of 0.5% to stabilize the movement of fluorescent beads by increasing the viscosity of the medium. The movement of beads under the tracheal epithelium was observed from the bottom of the dish using an inverted fluorescence microscope (Eclipse TE2000-U, Nikon, Tokyo, Japan) equipped with a CFI Plan Fluor objective lens (Nikon) and a CCD camera (Hamamatsu Photonics, Hamamatsu, Japan). The optimal configuration of the lens for tracking the movement of the beads, which were 0.5–2 mm from the chamber bottom, was × 20 magnification, an NA of 0.5, and a long working distance (2.1 mm). The velocity of each bead was calculated by dividing the width of the field of view by the time each individual bead took to travel across the field. Three or more independent tracheal samples and at least 10 fields of view in each sample were analyzed (more than 30 fields for each condition, 5 or more beads in each fields). The fluid movement velocity was measured from the migration distance and the time of travel for the beads by tracking individual fluorescent beads using Aquacosmos image analysis software (Hamamatsu Photonics). The rainbow trace was depicted with a macro provided for free software, ImageJ, from Hiratsuka laboratory of JAIST (https://www.jaist.ac.jp/ms/labs/hiratsuka/).

### Analysis of the ciliary beating orientation

The analysis was performed as described previously [13, 14] with slight modifications. After the tracheal tissue was transiently cultured in culture medium, the cilia tips of ciliated cells were labeled with Indian ink diluted with culture medium (1:100) to analyze intact cilia. The motility of ink-labeled cilia tips was recorded using HAS-L1 and HAS-U1 high-speed cameras (DITECT Co. Ltd, Tokyo, Japan) at 300 fps to reflect ciliary motility. The recording was performed at 23–28 °C. CBF was determined by subjecting the original traces to fast Fourier transform using Excel (Microsoft). The amplitude of ciliary beating, effective stroke velocity, recovery stroke velocity, and the effective stroke velocity/recovery stroke velocity ratio were calculated from the movement of the cilia tips. CBF data were presented as the median (range). The amplitude of ciliary beating, effective stroke velocity, recovery stroke velocity, and effective stroke velocity/recovery stroke velocity ratio were presented as the mean ± SEM. Three independent tracheal samples and at least 10 ink-labeled cilia in each sample were analyzed (n = more than 30 ink-labeled cilia for each condition). Kymographs of ciliary beating were depicted with a macro embedded in ImageJ.

### Adenosine triphosphate (ATP) measurements

ATP concentrations were measured in culture supernatants using an ATP assay kit based on luminometric techniques (Lucifell 250 plus, Kikkoman Biochemifa, Tokyo, Japan) according to the manufacturer’s protocol. In total, 100 µL of culture medium from tracheal tissue with or without polyI:C were used. Briefly, 100 µL of the ATP extraction reagent were added to each sample, and after 20 s, luciferin-luciferase (100 µL) was added to each sample. The luminescence of each sample was measured using Lumitester C-100 (Kikkoman Biochemifa).

### PolyI:C and suramin

PolyI:C and suramin were purchased from Sigma-Aldrich and used in this study at concentrations of 100 µg/mL and 100 µM, respectively.

### IAV infection

IAV strain A/Yokohama/110/2009 (H3N2) was provided by Dr. Kawakami (Yokohama City Inst. of Health, Japan). The median tissue culture infectious dose of the virus stock solution was 6 × 10^5^. The virus stock solution was diluted with DMEM containing sodium pyruvate solution up to 100-fold. Tracheal tissue taken from WT and TLR3-KO mice was infected with 2 mL of IAV solution for 1 h, and cilia-driven flow and ciliary beating orientation were analyzed. For inactivation of IAV, IAV in 20 µL medium was treated either with UV irradiation (254 nm; HL-2000 HybriLinker, Upland, CA) for 30 min.

### RNA isolation and real-time polymerase chain reaction (PCR)

After murine tracheal tissues were cultured with/without IAV for 1 h or 24 h, total RNA of the tracheae was extracted using the TRIzol reagent (Invitrogen, Carlsbad, CA, USA). For quantification of murine β-actin mRNA, reverse transcription (RT)-PCR was performed on 50 ng total RNA using the SuperScript VILO cDNA Synthesis Kit (Thermo Fisher Scientific, Rockford, IL, USA). Quantitative real-time PCR was conducted using the THUNDERBIRD SYBR qPCR Mix (TOYOBO, Osaka, Japan) on the CFX Connect Real-Time System (Bio-Rad, Hercules, CA, USA) according to manufacturer instruction. For quantification of IAV RNA, the one-step RT- quantitative real-time PCR method was performed in a 10 μL volume containing 1 μL of TaqMan Fast Virus 1-Step Master Mix (Thermo Fisher Scientific), 0.7 μL of forward primer IAV MP-39-67For (10 pmol/μL), 0.7 μL of reverse primer IAV MP-183-153Rev (10 pmol/μL), 0.5 μL of TaqMan probe IAV MP-96-75Probe (1 pmol/μL), 2 μL total RNA (20 ng), and 5.1 μL RNase-free water on the CFX Connect Real-Time System (Bio-Rad). Cycling conditions were as follows: reverse transcription at 50 °C for 5 min and 95 °C for 2 min, followed by 45 cycles of PCR at 95 °C for 15 s and 60 °C for 60 s. The IAV RNA levels were normalized to the levels of murine β-actin mRNA. The results were presented as 2^−(Ct of IAV RNA − Ct of β-actin)^ in arbitrary units. The sequences of primers and probe used in real-time RT-qPCR analysis were as follows:

murine β-actin For 5′-AGTGTGACGTTGACATCCGT-3′

murine β-actin Rev 5′-TGCTAGGAGCCAGAGCAGTA-3′

IAV MP-39–67 For 5′-CCMAGGTCGAAACGTAYGTTCTCTCTATC-3′

IAV MP-183–153 Rev 5′-TGACAGRATYGGTCTTGTCTTTAGCCAYTCCA-3′

IAV MP-96–75 Probe 5′-[FAM]-ATYTCGGCTTTGAGGGGGCCTG-[BHQ1]-3′

### Statistical analysis

Data are presented as the mean ± SEM, unless otherwise noted. Comparisons between groups were performed using Student’s t-test. *p* ≤ 0.05 was considered statistically significant.

## Results

### IAV promotes cilia-driven flow and ciliary activity in the tracheal epithelium

To confirm IAV infectivity in organ culture of WT murine tracheal tissues, IAV RNA level were evaluated using real-time PCR. IAV RNA was detected in tracheal tissues cultured with IAV for 1 h, whereas no IAV RNA was detected in tracheal tissues cultured without IAV (Additional file 5: Fig. S1). The IAV RNA levels were significantly higher in the tracheal tissues cultured with IAV for 24 h than those for 1 h (Additional file 5: Fig. S1). These results indicated that tracheal tissues were productively infected with IAV after transient organ culture with IAV solution.

**Fig. 1:**
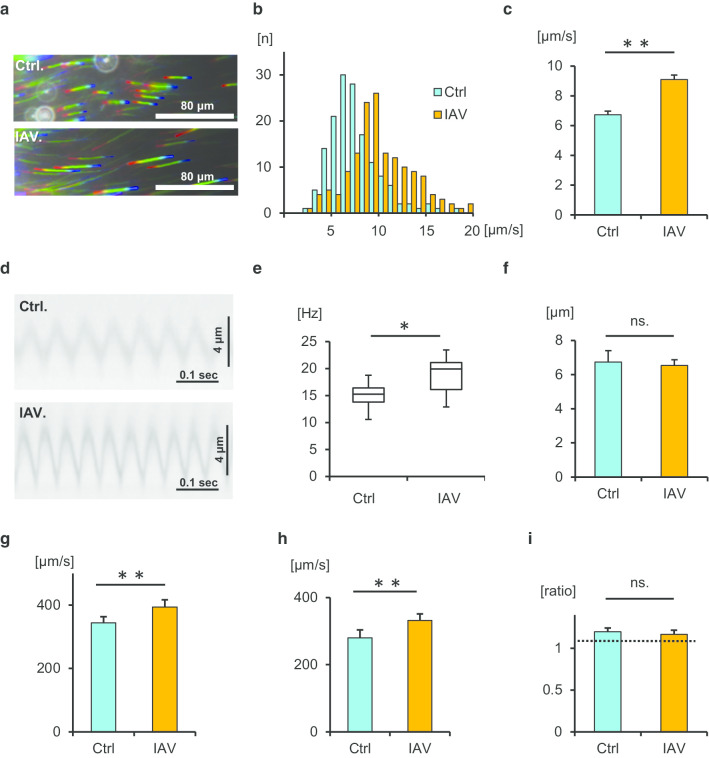
Impact of influenza A virus (IAV) infection on cilia-driven flow and ciliary beating orientation. Wild-type (WT) murine tracheal epithelia were incubated for 1 h with/without IAV in the culture medium, and then cilia-driven flow and ciliary beating orientation were evaluated. **a** Representative beads trajectories of cilia-driven flow (rainbow trace for 4.4 s). **b** and **c** Both the histogram (**b**) and bar chart (**c**) of cilia-driven flow demonstrated that IAV infection significantly increased cilia-driven flow (Ctrl, 6.72 ± 0.25 µm/s; IAV, 9.10 ± 0.29 µm/s; n = 150 beads in each condition). **d** Kymographs of ciliary beating with/without IAV infection. **e** IAV infection significantly increased ciliary beat frequency (CFB; Ctrl, 14.94 [10.55–18.75] Hz; IAV, 19.92 [12.89–23.44] Hz; n = 30 each). **f** The amplitude of ciliary beating did not differ between the conditions (Ctrl, 6.74 ± 0.66 µm; IAV, 6.54 ± 0.33 µm). **g** and **h** The effective stroke velocity (**g**) (Ctrl, 344.2 ± 19.5 µm/s; IAV, 394.0 ± 23.0 µm/s) and recovery stroke velocity (**h**) (Ctrl, 279.8 ± 23.8 µm/s; IAV, 331.9 ± 19.2 µm/s) were significantly increased by IAV infection compared with the Ctrl levels. **i** The ratio of effective stroke velocity to recovery stroke velocity was not different between the two conditions (Ctrl, 1.20 ± 0.05; IAV, 1.17 ± 0.05). *p < 0.01, **p < 0.05. *Ctrl* control, *ns* not significant. CBF data were presented as the median (range)

Initially, we investigated whether short-term IAV infection (1 h) can affect ciliary activity and mucociliary clearance in the tracheal epithelium. We recorded and analyzed cilia-driven flow over the tracheal surface using small polystyrene beads in the presence or absence of IAV infection (Additional files 1, 2). Representative beads trajectories are presented in Fig. 1a. As shown in Fig. 1b, c, IAV infection significantly increased cilia-driven flow compared with the control level. Ciliary motility was recorded using a microscope equipped with a high-speed digital camera (Additional files 3, 4). Kymographs of the movies in the presence or absence of IAV infection are shown in Fig. 1d. An analysis of the ciliary beating orientation revealed that CBF was significantly increased by IAV infection compared with the control findings (Fig. 1e). The amplitude of ciliary beating did not differ between the conditions (Fig. 1f). Both the effective stroke velocity and recovery stroke velocity were significantly greater in the presence of IAV than in its absence (Fig. 1g, h). The effective stroke velocity/recovery stroke velocity ratio did not differ between the conditions (Fig. 1i). Taken together, our findings indicate that short-term IAV infection promotes cilia-driven flow and ciliary activity in the airway epithelium.

Moreover, tracheal tissues were cultured with UV-inactivated IAV for 1 h, and then IAV RNA levels and cilia-driven flows were evaluated. IAV RNA levels were quite low in tracheal culture with UV-inactivated IAV (Additional file 5: Fig. S2A), confirming the loss of infectivity of UV-inactivated IAV. IAV-mediated increase of cilia-driven flow was abolished by UV irradiation (Additional file 5: Fig. S2B), suggesting that IAV infection, not mediators in the viral solution, increased cilia-driven flow in the airway epithelium.

### TLR3 is involved in IAV-promoted cilia-driven flow and ciliary activity

**Fig. 2:**
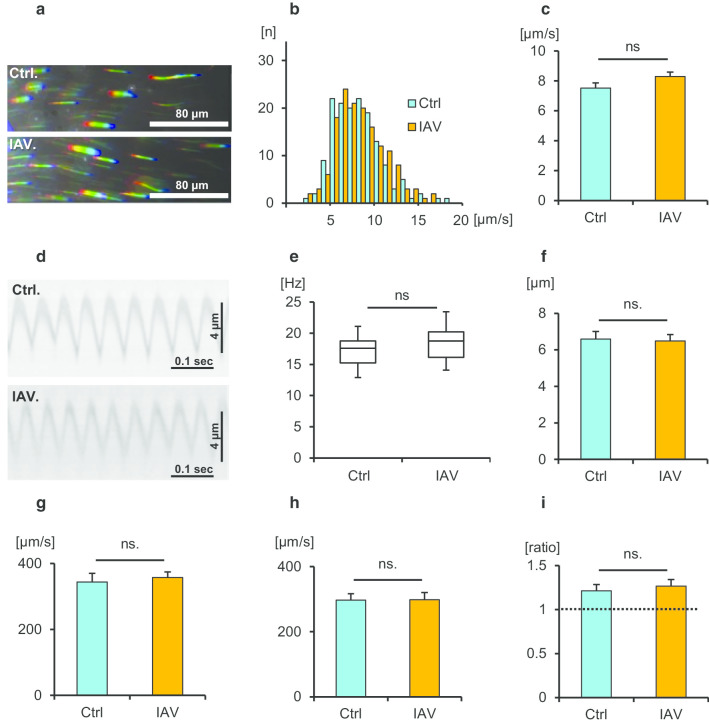
Involvement of Toll-like receptor 3 (TLR3) in influenza A virus (IAV)-mediated cilia-driven flow and ciliary beating orientation. TLR3-KO murine tracheal epithelia were incubated for 1 h with or without IAV in the culture medium, and then cilia-driven flow and ciliary beating orientation were evaluated. **a** Representative beads trajectories of cilia-driven flow (rainbow trace for 4.4 s). **b** and **c** Both the histogram (**b**) and bar chart (**c**) of cilia-driven flow demonstrated that IAV infection had no impact of cilia-driven flow in TLR-3KO culture (Ctrl, 7.51 ± 0.35 µm/s; IAV, 8.28 ± 0.30 µm/s; n = 150 beads in each condition). **d** Kymograph of ciliary beating with or without IAV infection. **e** IAV infection did not affect ciliary beat frequency (CBF) in TLR3-KO culture (Ctrl, 17.58 [12.89–21.09] Hz; IAV, 18.75 [14.06–23.44] Hz; n = 30 in each condition). **f** Amplitude of ciliary beating was similar between the two conditions (Ctrl, 6.59 ± 0.41 µm; IAV, 6.48 ± 0.35 µm). **g** and **h** The effective stroke velocity (**g**) (Ctrl, 343.6 ± 26.5 µm/s; IAV, 357.3 ± 17.1 µm/s) and recovery stroke velocity (**h**) (Ctrl, 296.9 ± 19.9 µm/s; IAV, 298.2 ± 21.9 µm/s) did not differ between the groups. **i** The ratio of the effective stroke velocity to recovery stroke velocity was not different between the two conditions (Ctrl, 1.21 ± 0.07; IAV, 1.26 ± 0.08). *Ctrl* control, *ns* not significant; CBF data were presented as the median (range)

**Fig. 3:**
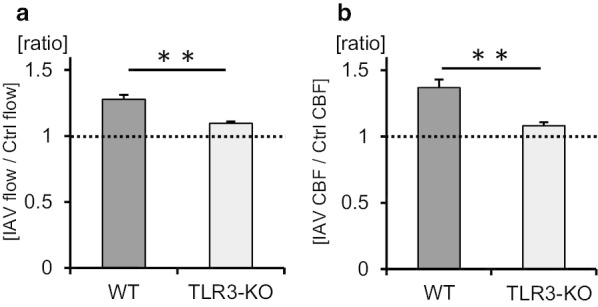
The ratio of cilia-driven flow and CBF between IAV infection and control in WT mice and TLR3-KO mice. **a** The ratio of cilia-driven flow between IAV infection and Ctrl was significantly greater in WT mice than in TLR3-KO mice (WT, 1.28 ± 0.04; TLR3-KO, 1.10 ± 0.01; n = 4 independent experiments). **b** The ratio of CBF between IAV infection and Ctrl was also greater in WT mice that in TLR3-KO mice (WT, 1.37 ± 0.06; TLR3-KO, 1.08 ± 0.03; n = 4 independent experiments). **p < 0.05. *Ctrl* control, *WT* wild-type, *KO* knockout

Previous studies demonstrated that the influenza virus induces the antiviral cytokines IFN-β and IFN-λ via TLR3 activation in airway epithelial cells [25, 30]. To determine whether TLR3 signaling was involved in IVA-promoted ciliary activity, the tracheal tissues of TLR3-KO mice were used. Cilia-driven flow and ciliary motility in TLR3-KO culture were recorded and analyzed. Representative beads trajectories are presented in Fig. 2a. As shown in Fig. 2b, c, IAV infection had no impact on cilia-driven flow in the tracheal epithelium of TLR3-KO mice. Kymographs of the movies of ciliary motility are shown in Fig. 2d. CBF was not increased by IAV infection compared with its control level in TLR3-KO tracheal epithelium (Fig. 2e). The amplitude of ciliary beating did not differ between the two conditions (Fig. 2f). No differences were observed in the effective stroke velocity, recovery stroke velocity, and effective stroke velocity/recovery stroke velocity ratio between the two conditions (Fig. 2g–i). IAV infectivity were not different between WT culture and TLR3-KO culture, since the IAV RNA levels in TLR3-KO tracheae cultured with IAV for 1 h were nearly identical to those in WT tracheae cultured with IAV (Additional file 5: Fig. S3). The ratio of cilia-driven flow between IAV infection and the control was significantly greater in WT mice than in TLR3-KO mice (Fig. 3a). The ratio of CBF between IAV infection and the control was also greater in WT mice than in TLR3-KO mice (Fig. 3b). These results confirmed that TLR3 signaling was essential to elicit an increase of cilia-driven flow and CBF in response to IAV infection in the airway epithelium.

### PolyI:C increases cilia-driven flow and ciliary activity via TLR3 signaling

**Fig. 4:**
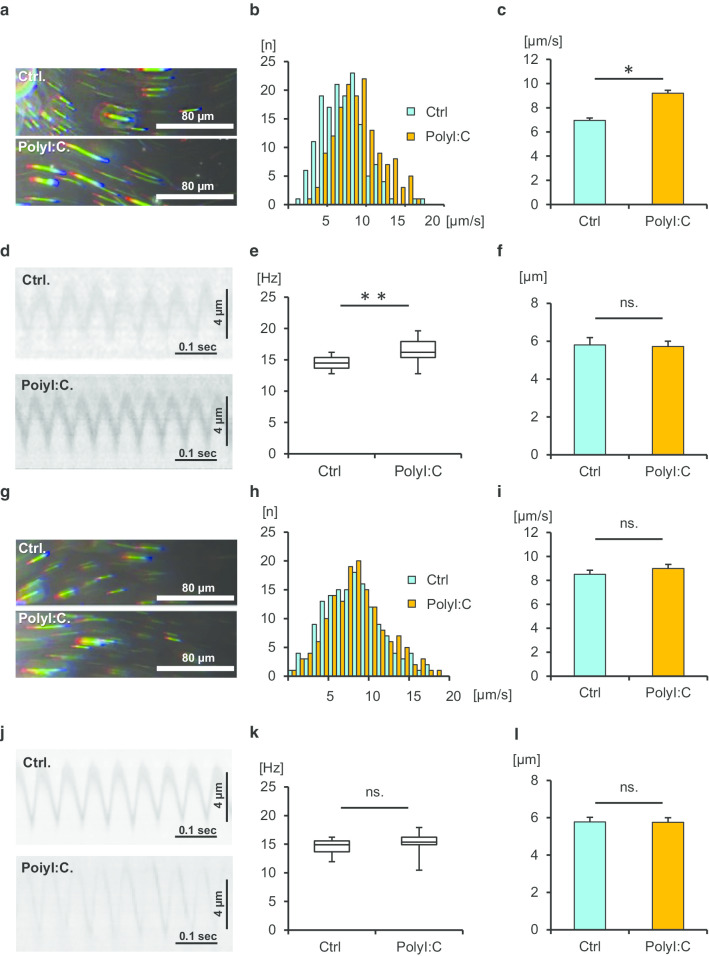
PolyI:C increases cilia-driven flow and ciliary activity via Toll-like receptor 3 (TLR3) signaling. WT and TLR3-KO murine tracheal epithelia were incubated for 30 min with or without polyI:C in the culture medium, and then cilia-driven flow and ciliary beat frequency (CBF) were evaluated. **a** Representative beads trajectories of cilia-driven flow in WT culture (rainbow trace for 4.4 s). **b** and **c** Both the histogram (**b**) and bar chart (**c**) of cilia-driven flow demonstrated that polyI:C treatment significantly increased cilia-driven flow in WT culture (Ctrl, 6.96 ± 0.21 µm/s; polyI:C, 9.20 ± 0.23 µm/s; n = 150 beads in each condition). **d** Kymographs of ciliary beating in WT culture. **e** PolyI:C treatment significantly increased CFB in WT culture (Ctrl, 14.51 [12.80–16.21] Hz; polyI:C, 16.21 [12.80–19.62] Hz; n = 30 in each condition). **f** The amplitude of ciliary beating did not differ between the two conditions (Ctrl, 5.80 ± 0.38 µm; polyI:C, 5.72 ± 0.28 µm). **g** Representative beads trajectories of cilia-driven flow in TLR3-KO culture (rainbow trace for 4.4 s). **h** and **i** Both the histogram (**h**) and bar chart (**i**) of cilia-driven flow demonstrated that polyI:C treatment had no impact on cilia-driven flow in TLR-KO culture (Ctrl, 8.50 ± 0.34 µm/s; polyI:C, 8.99 ± 0.34 µm/s; n = 150 beads in each condition). **j** Kymographs of ciliary beating in TLR3-KO culture. **k** PolyI:C treatment did not affect CBF in TLR3-KO culture (Ctrl, 14.92 [11.95–16.21] Hz; polyI:C, 15.36 [10.45–17.92] Hz; n = 30 in each condition). **l** The amplitude of ciliary beating was not different between the conditions (Ctrl, 5.78 ± 0.25 µm; polyI:C, 5.76 ± 0.24 µm). *p < 0.01, **p < 0.05. *Ctrl* control, *ns* not significant. CBF data were presented as the median (range)

**Fig. 5:**
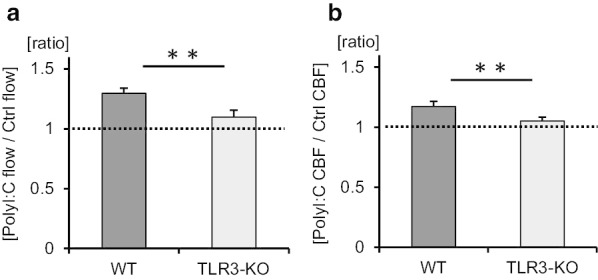
The ratio of cilia-driven flow and CBF between polyI:C treatment and control in WT mice and TLR3-KO mice. **a** The ratios of cilia-driven flow between polyI:C treatment and Ctrl were significantly greater in WT mice than in TLR3-KO mice (WT, 1.30 ± 0.04; TLR3-KO, 1.10 ± 0.06; n = 4 independent experiments). **b** The ratios of CBF between polyI:C treatment and Ctrl were significantly greater in WT mice than in TLR3-KO mice (WT, 1.17 ± 0.04; TLR3-KO, 1.05 ± 0.03; n = 4 independent experiments). **p < 0.05. *Ctrl* control, *WT* wild-type, *KO* knockout

We next aimed to determine whether TLR3 activation is sufficient to enhance cilia-driven flow and ciliary activity in the tracheal epithelium. The tracheal tissues of WT mice were cultured with or without polyI:C, a ligand of TLR3, for 30 min, and cilia-driven flow and ciliary motility were recorded. The representative beads trajectories are shown in Fig. 4a. We found that polyI:C treatment for 30 min significantly increased cilia-driven flow compared with the control level in WT culture (Fig. 4b–c). Kymographs of ciliary motility are shown in Fig. 4d. CBF was significantly increased by polyI:C treatment compared with the control level in WT culture (Fig. 4e). The amplitude of ciliary beating was not changed by polyI:C treatment (Fig. 4f). To confirm the involvement of TLR3 in polyI:C-mediated increases of ciliary activity, the tracheal tissues of TLR3-KO mice were incubated with or without polyI:C, and cilia-driven flow and CBF were evaluated. Representative beads trajectories and kymographs of ciliary motility in TLR3-KO culture are shown in Fig. 4g, j. As shown in Fig. 4h, i, polyI:C treatment had no impact on cilia-driven flow in the TLR3-KO tracheal epithelium. CBF was not increased by polyI:C treatment compared with the control level in TLR3-KO culture (Fig. 4k). The amplitude of ciliary beating was similar under the two conditions (Fig. 4l). As show in Fig. 5a, the ratio of cilia-driven flow between polyI:C treatment and the control was significantly greater in WT mice than in TLR3-KO mice. Similarly, the ratio of CFB between polyI:C treatment and the control was greater in WT mice than in TLR3-KO mice (Fig. 5b). These results confirmed that TLR3 activation was sufficient to enhance cilia-driven flow and ciliary activity in the airway epithelium.

### TLR3 activation mediates ATP release in the tracheal epithelium

**Fig. 6:**
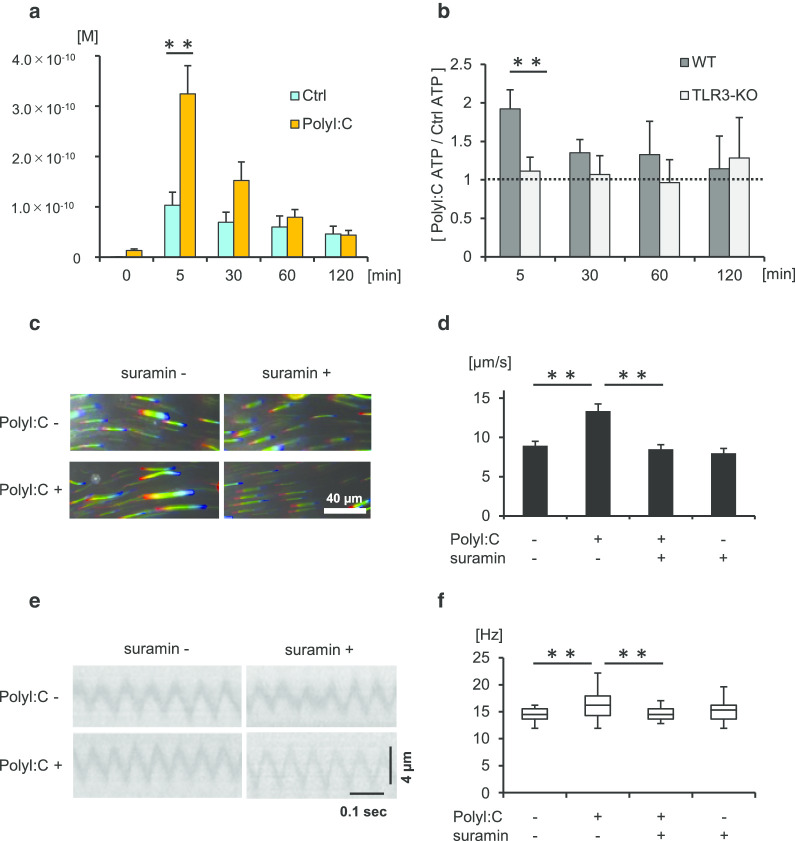
Toll-like receptor 3 (TLR3)-mediated ciliary activation was dependent on extracellular adenosine triphosphate (ATP) release and the autocrine ATP-purinergic P2 receptor (P2R) loop. **a** PolyI:C treatment significantly increased ATP concentrations in the culture supernatant after 5 min versus the Ctrl findings in WT culture (Ctrl, 1.03 × 10^−10^ ± 2.56 × 10^−11^ M; PolyI:C, 3.24 × 10^−10^ ± 5.61 × 10^−11^ M; n = 4 each). **b** The ATP concentration ratios (polyI:C ATP/Ctrl APT) at 5 min was significantly greater in WT culture than in TLR3-KO culture (WT, 1.92 ± 0.25; TLR3-KO, 1.11 ± 0.18; n = 4). WT murine tracheal epithelia were treated with/without polyI:C and/or suramin for 30 min, and cilia-driven flow and ciliary beat frequency (CBF) were evaluated. **c** Representative beads trajectories in each condition (rainbow trace for 4.4 s). **d** Suramin suppressed polyI:C-promoted cilia-driven flow to the Ctrl levels, but it did not affect basal ciliary driven flow (Ctrl, 8.95 ± 0.14 µm/s; polyI:C, 13.36 ± 0.24 µm/s; polyI:C + suramin, 8.49 ± 0.15 µm/s; suramin, 7.98 ± 0.15 µm/s; n = 150 beads in each condition). **e** Kymographs of the movies under each condition. **f** Suramin inhibited the polyI:C-mediated increase of CBF, but it did not affect basal CBF (Ctrl, 14.51 [11.95–16.21] Hz; polyI:C, 16.21 [11.95–22.19] Hz; polyI:C + suramin, 14.51 [12.80–17.07] Hz; suramin, 15.36 [11.95–19.63] Hz; n = 30 in each condition] **p < 0.05. *Ctrl* control, *WT* wild-type, *KO* knockout. CBF data were presented as the median (range)

Extracellular ATP is known to increase CBF and hasten mucociliary clearance in the airway by activating purinergic P2 receptors (P2R) signaling [31, 32]. Thus, we next evaluated whether TLR3 activation induced extracellular ATP release in tracheal tissue. ATP concentrations were measured in the culture supernatants with or without polyI:C treatment for 5–120 min. As shown in Fig. 6a, polyI:C treatment significantly increased ATP levels at 5 min compared with the control in WT culture. The ratios of ATP concentrations between polyI:C treatment and the control in WT and TLR3-KO culture are shown in Fig. 6b. The ATP concentration ratios (polyI:C ATP/control ATP) at 5 min were significantly greater in WT culture than in TLR3-KO culture.

### ATP released by TLR3 activation increased cilia-driven flow and CBF via the ATP-P2R loop

To determine whether extracellular ATP release following TLR3 activation can promote ciliary activity and transport via P2R, the potent P2R antagonist suramin was added together with polyI:C to block ATP-P2R binding, and cilia-driven flow and ciliary motility were recorded and evaluated. Representative beads trajectories in each condition with/without polyI:C and suramin are shown in Fig. 6c. Suramin significantly suppressed polyI:C-promoted cilia-driven flow to the control level (Fig. 6d). Suramin had no effect on basal cilia-driven flow. Kymographs of the movies under each condition are shown in Fig. 6e. The addition of suramin significantly inhibited the polyI:C-mediated increase of CBF, but it did not affect basal CBF (Fig. 6f). To confirm that extracellular ATP contributes to increase of ciliary activity in organ culture of murine trachea, ATP was added to culture media and CBF were evaluated. ATP stimulation for 5 min significantly increased CBF in WT culture as compared with control (Additional file 5: Fig. S4). Taken together, our findings indicate that extracellular ATP release by TLR3 activation increases cilia driven flow and CBF via autocrine ATP-P2R loop in the airway epithelium.

## Discussion

In the present study, we investigated the impact of respiratory virus infection on mucociliary clearance, with a particular focus on ciliary activity and cilia-driven flow, using organ culture models of murine trachea and in vitro IAV infection models. This study provides evidence that IAV infection in the airway readily stimulates ciliary activity and ciliary transport via TLR3 activation, which promote mucociliary clearance to hasten the elimination of viruses from the respiratory tract, highlighting their important roles as initial airway defense responses.

To the best of our knowledge, this is the first report to reveal that IAV directly promotes cilia-driven flow and ciliary activity in the airway epithelium. During respiratory viral infections, airway epithelial cells play central roles in airway host defenses by producing antiviral substances through the recognition of virus nucleic acid by PRRs, including TLR3 [8, 24–28]. However, the direct effects of viral infection on ciliary activity in the airway epithelium had not been studied previously. Our results illustrated that IAV infection immediately increased cilia-driven flow and ciliary activity in the airway epithelium. To minimize airway epithelium impairment during viral infection, invading viruses should be eliminated from the respiratory tract as soon as possible. Thus, it is reasonable that airway epithelial cells have the ability to promote ciliary activity immediately in response to virus invasion, which hastens the elimination of the virus from the airway as an initial host defense response. A prior study using in vivo models of IAV infection in the chinchilla Eustachian tube demonstrated that IAV infection induced a decrease of CBF in the Eustachian tube epithelium 7–14 days after infection via the impairment of cilia and ciliated cells [33]; however, they recorded no data regarding changes of ciliary activity within 1 day after IAV infection. Moreover, no study reported the direct effects of short-term IAV infection on the ciliary activity of airway epithelial cells. The methodology used in this study, organ cultures of murine trachea with short-term IAV infection and imaging techniques to analyze ciliary motion and cilia-driven flow, enabled us to investigate the impact of short-term IAV infection on airway ciliary activity. Moreover, these findings should spur future research to clarify whether promotion of ciliary activity can be targeted using drugs to hasten to elimination of the virus from the airway.

The present study also revealed the potential roles of TLR3 signaling as a regulator of ciliary activity in the airway epithelium. The regulatory effects of TLR signaling activation on ciliary activity and mucociliary clearance in the respiratory tract had not been clarified. In the present study, we mainly focused on TLR3 using IAV infection models and polyI:C stimulation, since we previously observed that TLR3 signaling played pivotal roles in initial antiviral responses to induce the expression of IFN-λ and proinflammatory cytokines and chemokines (e.g., G-CSF, IL-8, IL-17C, CXCL1, CXCL5) in normal human bronchial epithelial cells [26–29]. Our results in this study revealed that TLR3 activation increased ciliary activity and cilia-driven flow in the airway epithelium. The involvement of TLR3 signaling in IAV- and polyI:C-mediated increases of ciliary activity was confirmed using TLR3-KO tracheal culture. Given that TLR signaling plays a vital role in the initiation of host defense responses in the airway epithelium, it is understandable that TLR signaling increases ciliary activity and cilia-driven flow. A former study using an experimental murine model of IAV infection demonstrated that TLR3-KO mice had a higher IAV amount in their lung as compared to WT mice [34], suggesting that TLR3 signaling was involved in viral clearance. In addition, Our findings provide insight into the novel functions of TLR3 signaling as a regulator of mucociliary clearance, a primary defense mechanism for protecting the airways against harmful viral and bacterial pathogens.

Few studies have examined the effects of TLRs, excluding TLR3, on ciliary function in airway epithelial cells. Alpizar et al. reported that lipopolysaccharides (LPS), which are ligands of TLR4, increased CBF in mouse tracheobronchial epithelial cells by activating transient receptor potential vanilloid 4 (TRPV4) cation channels in a process that was independent of the TLR4 signaling pathway [35]. They also mentioned that LPS-induced TRPV4 activation occurred more rapidly than activation of the canonical TLR4 immune pathway. In the present study, we first found that IAV- or polyI:C-mediated increases of ciliary activity were dependent on TLR3 signaling. In addition, 1 h of IAV exposure or 30 min of polyI:C stimulation were sufficient to increase CBF and cilia-driven flow, confirming that this TLR3-mediated ciliary activation occurred more rapidly than the TLR3-mediated upregulation of antiviral components (e.g., IFN-λ, IFN-β, IL-8, IL-17C, G-CSF) reported in our previous studies [26–28]. Moreover, TLR3 activation readily provoked extracellular ATP release, which resulted in subsequent increases in ciliary function via the ATP-P2R pathway. Taken together, TLR3 expressed in airway epithelial cells rapidly recognizes respiratory viruses and mediates antiviral host defense responses through a variety of mechanisms (e.g., ATP release, increases of ciliary activity and gene expression) to suppress viral invasion and replication.

It has been reported that airway epithelial cells can release exocytic ATP under cellular deformation and mechanical stimulations [36–39], which in turn increased ciliary activity via the autocrine activation of P2Rs, including P2Y receptors. Contrarily, only a few studies have mentioned the activation of TLR3-triggered ATP release in epithelial cells [40, 41]. It also remains unclear whether TLR3-mediated ATP release is involved in the activation of ciliary function in airway epithelial cells. Our present study demonstrated that TLR3 activation in the tracheal epithelium increased the extracellular ATP concentration, accompanied by increases of cilia-driven flow and CBF in WT cultures but not in TLR3 KO cultures. In addition, blockade of P2R signaling using suramin suppressed polyI:C-mediated increases of cilia-driven flow and CBF, confirming the important role of TLR3-mediated ATP release in the dynamic regulation of airway ciliary function. It is hard to rule out the possibility that multiple pathways may play roles in TLR3-mediated increase of cilia-driven flow and CBF; however, extracellular ATP release and autocrine ATP-P2R loop may be major mechanisms, since blockade of ATP-P2R binding by suramin suppressed polyI:C-promoted cilia-driven flow and CBF to the control levels.

The intracellular mechanisms underlying extracellular ATP release induced by TLR3 activation in the airway epithelium have not been fully elucidated. Pannexins and connexins are structural components of gap junctions that form hemichannels, which permit the passage of ion and other small molecules including ATP, between the intra- and extracellular compartments [42–44]. In the airway epithelium, extracellular ATP can be released by a conductive mechanism mediated by pannexins and connexins [39, 45, 46], which results in increased CBF via P2R activation. Recently, calcium homeostasis modulator 1 (CALHM1), a transmembrane protein that shares structural features with connexins and pannexins, is known to participate in ATP release and the modulation of ciliary activity following mechanical stimulation in airway epithelial cells [47]. These transmembrane proteins (e.g., pannexins, connexins, CALHM1) may be involved in the extracellular ATP release driven by TLR3 activation, which will be of great interest in future studies. Further research is warranted to elucidate the detailed molecular mechanisms of TLR3-mediated ATP release in airway epithelial cells.

## Conclusions

This study focused on early changes on ciliary activity and cilia-driven flow after short-term IAV infection. Our data provide novel findings that short-term IAV infection stimulates ciliary activity and increases cilia-driven flow and CBF in the airway epithelium via TLR3 signaling. Mechanical analyses revealed that extracellular ATP release in response to TLR3 activation promoted ciliary activity through autocrine ATP-P2R loop. Proper ciliary function is required for effective mucociliary clearance to protect the airways against microorganism and contaminants. In the presence of respiratory viral infection, prompt cellular responses are critical for eliminating the invading viruses and inducing antiviral immunity. From this viewpoint, rapid activation of airway ciliary function in response to IAV infection is an essential regulatory mechanism of mucociliary clearance as an initial host defense response against viral respiratory infection.

## Supplementary information

**Additional file 1.** Cilia-driven flow over the tracheal surface using small polystyrene beads under the normal contiditon (control) in WT tracheal epithelium.

**Additional file 2.** Cilia-driven flow under the contiditon with IAV infection for 1h in WT tracheal epithelium, which was faster than that of control.

**Additional file 3.** Ciliary motility under the normal contiditon (control) in WT tracheal epithelium.

**Additional file 4.** Ciliary motility under the contiditon with IAV infection for 1h in WT tracheal epithelium.

**Additional file 5: Fig. S1.** IAV RNA levels in organ culture of murine tracheal tissues. **Fig. S2.** Inactivation of IAV by UV irradiation. **Fig. S3.** IAV RNA levels in tracheal tissues of WT and TLR3-KO mice. **Fig. S4.** Changes of CBF by ATP stimulation.

## Data Availability

The datasets used and/or analyzed during the current study are available from the corresponding author on reasonable request.

## Abbreviations

ATP: Adenosine triphosphate
CALHM1: Calcium homeostasis modulator 1
CBF: Ciliary beat frequency
IAV: Influenza A virus
IFN: Interferon
IL: Interleukin
KO: Knockout
LPS: Lipopolysaccharides
P2R: P2 receptors
PRR: Pattern-recognition receptors
TLR: Toll-like receptor
TNF: Tumor necrosis factor
TRPV4: Transient receptor potential vanilloid 4
WT: Wild-type
