# Supplementary material for: Influenza A virus enhances ciliary activity and mucociliary clearance via TLR3 in airway epithelium
Source: Respir Res. 2020 Oct 27;21:282. doi: 10.1186/s12931-020-01555-1 (PMC7590254; doi:10.1186/s12931-020-01555-1)
Supplement: Supplementary file 5 — Additional file 5: Fig. S1. IAV RNA levels in organ culture of murine tracheal tissues. Fig. S2. Inactivation of IAV by UV irradiation. Fig. S3. IAV RNA levels in tracheal tissues of WT and TLR3-KO mice. Fig. S4. Changes of CBF by ATP stimulation. [file 12931_2020_1555_MOESM5_ESM.docx]

**Additional file5. (Figure S1-S4)**

**Figure S1.**


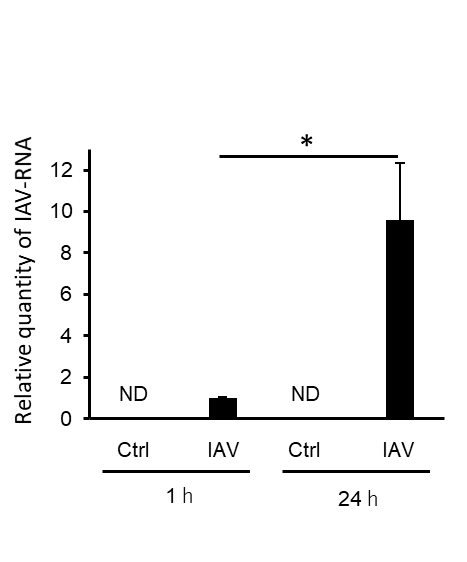


**Figure S1. IAV RNA levels in organ culture of murine tracheal tissues**

WT murine trachea tissues were cultured with/without IAV for 1 h or 24 h. IAV RNA levels were determined by reverse transcription real-time PCR (RT-qPCR) and normalized to β-actin mRNA. The IAV RNA levels were significantly higher in the tracheal tissues cultured with IAV for 24 h than those for 1 h (IAV 1h, 1.00 ± 0.03; IAV 24h, 9.56 ± 2.79; n = 3 in each condition). No IAV RNA was detected in tracheal tissues cultured without IAV. * *p* < 0.01, Ctrl, control; ND not detected

**Figure S2.**


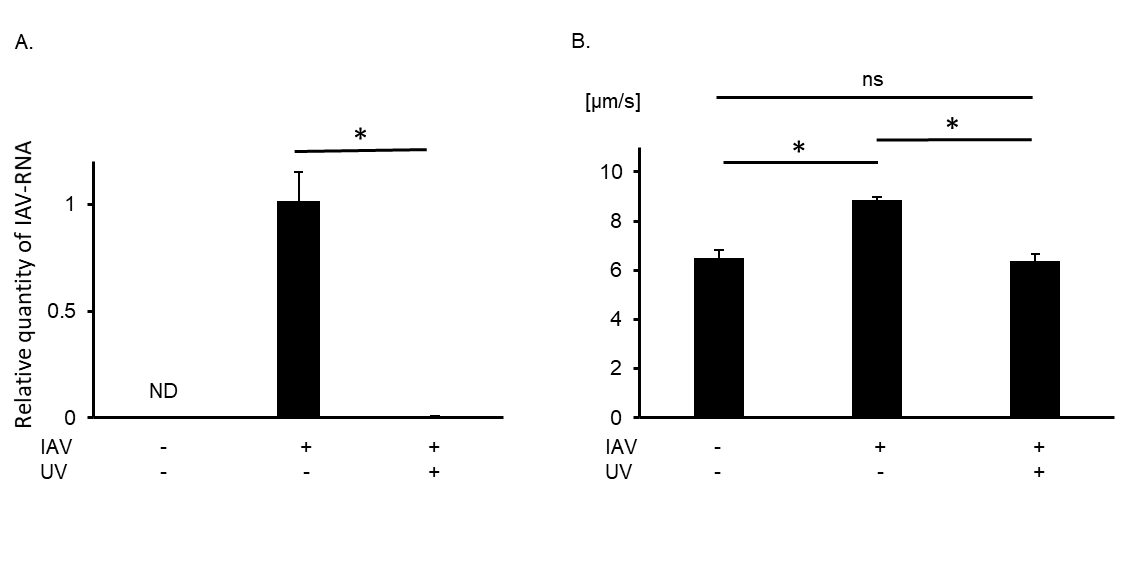


**Figure S2. Inactivation of IAV by UV irradiation**

For inactivation of IAV, IAV in 20 µL medium was treated either with UV irradiation (254 nm; HL-2000 HybriLinker, Upland, CA) for 30 min.

1. IAV RNA levels were quite low in tracheal tissues cultured with UV-inactivated IAV as compared to those with IAV (IAV, 1.00 ± 0.14; UV-IAV, 0.01 ± 0.004; n = 3 in each condition).
2. IAV-mediated increase of cilia-driven flow was abolished by UV irradiation (Ctrl, 6.51 ± 0.31 µm/s; IAV, 8.85 ± 0.26 µm/s; UV-IAV, 6.38 ± 0.12 µm/s; n = 150 beads in each condition).

* *p* < 0.01. ND, not detected; ns, not significant.

**Figure S3.**


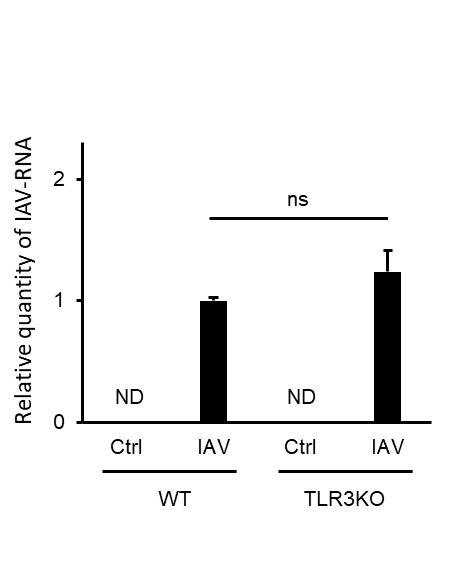


**Figure S3. IAV RNA levels in tracheal tissues of WT and TLR3-KO mice**

IAV RNA levels in TLR3-KO tracheal tissue cultured with IAV were nearly identical to those in WT with IAV. No IAV RNA was detected in WT and TLR3-KO tracheae without IAV (WT IAV, 1.00 ± 0.03; TLR3-KO IAV, 1.21 ± 0.18; n = 3 in each condition)

Ctrl, control; ND, not detected; ns, not significant.

**Figure S4.**


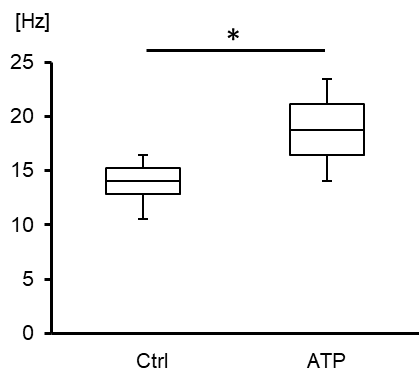


**Figure S4. Changes of CBF by ATP stimulation**

ATP stimulation (final concentration: 1 x 10^-8^ M) for 5 min significantly increased CBF in WT culture as compared with control [Ctrl 14.06 (10.55 – 16.41) Hz; ATP 18.75 (14.06 – 23.44) Hz, n = 20 in each condition].

* *p* < 0.01, CBF, ciliary beat frequency; Ctrl, control; ATP, Adenosine triphosphate. CBF data were presented as the median (range).
